# Low testosterone levels relate to poorer cognitive function in women in an *APOE*-ε4-dependant manner

**DOI:** 10.1186/s13293-024-00620-4

**Published:** 2024-06-05

**Authors:** Melanie A. Dratva, Sarah J. Banks, Matthew S. Panizzon, Douglas Galasko, Erin E. Sundermann, for the Alzheimer’s Disease Neuroimaging Initiative

**Affiliations:** 1 Department of Neurosciences, University of California, San Diego, 9500 Gilman Dr., La Jolla, CA 92093 USA; 2 Department of Psychiatry, University of California, San Diego, 9500 Gilman Dr., La Jolla, CA 92093 USA; 3 Center for Behavior Genetics of Aging, University of California, San Diego, 9500 Gilman Dr., La Jolla, CA 92092 USA; 4 UCSD ACTRI Building, 2W502-B8, 9452 Medical Center Drive (MC-0841), La Jolla, CA 92037 USA

**Keywords:** Testosterone, Alzheimer’s disease, Cognition, Sex differences, *APOE*-ε4

## Abstract

**Background:**

Past research suggests that low testosterone levels relate to poorer cognitive function and higher Alzheimer’s disease (AD) risk; however, these findings are inconsistent and are mostly derived from male samples, despite similar age-related testosterone decline in females. Both animal and human studies demonstrate that testosterone’s effects on brain health may be moderated by apolipoprotein E ε4 allele (*APOE*-ε4) carrier status, which may explain some previous inconsistencies. We examined how testosterone relates to cognitive function in older women versus men across healthy aging and the AD continuum and the moderating role of *APOE*-ε4 genotype.

**Methods:**

Five hundred and sixty one participants aged 55–90 (155 cognitively normal (CN), 294 mild cognitive impairment (MCI), 112 AD dementia) from the Alzheimer’s Disease Neuroimaging Initiative (ADNI), who had baseline cognitive and plasma testosterone data, as measured by the Rules Based Medicine Human DiscoveryMAP Panel were included. There were 213 females and 348 males (self-reported sex assigned at birth), and 52% of the overall sample were *APOE*-ε4 carriers. We tested the relationship of plasma testosterone levels and its interaction with *APOE*-ε4 status on clinical diagnostic group (CN vs. MCI vs. AD), global, and domain-specific cognitive performance using ANOVAs and linear regression models in sex-stratified samples. Cognitive domains included verbal memory, executive function, processing speed, and language.

**Results:**

We did not observe a significant difference in testosterone levels between clinical diagnostic groups in either sex, regrardless of *APOE*-ε4 status. Across clinical diagnostic group, we found a significant testosterone by *APOE*-ε4 interaction in females, such that lower testosterone levels related to worse global cognition, processing speed, and verbal memory in *APOE*-ε4 carriers only. We did not find that testosterone, nor its interaction with *APOE*-ε4, related to cognitive outcomes in males.

**Conclusions:**

Findings suggest that low testosterone levels in older female *APOE*-ε4 carriers across the aging-MCI-AD continuum may have deleterious, domain-specific effects on cognitive performance. Although future studies including additional sex hormones and longitudinal cognitive trajectories are needed, our results highlight the importance of including both sexes and considering *APOE*-ε4 carrier status when examining testosterone’s role in cognitive health.

**Supplementary Information:**

The online version contains supplementary material available at 10.1186/s13293-024-00620-4.

## Highlights

- We investigated the relationship between testosterone levels and cognitive function in women and men across the Alzheimer’s dementia continuum and the modifying role of the *APOE*-ε4 allele.
- A significant testosterone by *APOE*-ε4 interaction indicated that lower plasma testosterone levels related to worse global and domain-specific cognition, for verbal memory and processing speed, in female *APOE*-ε4 carriers only.
- Plasma testosterone levels did not relate to global or domain-specific cognition in males, regardless of *APOE*-ε4 status.
- While most studies examine the testosterone and brain health connection in men, our findings suggest that it is equally important, if not more, to examine this connection in women and to account for *APOE*-ε4 status.
- Upon replication, our findings suggest the potential for low testosterone levels as a modifiable risk factor in which intervention approaches can be optimized for each sex.

**Supplementary Information:**

The online version contains supplementary material available at 10.1186/s13293-024-00620-4.

## Abstract (plain language summary)

Sex differences often suggest a role of sex hormones, and in Alzheimer’s Disease (AD) research, women show higher disease prevalence, accelerated cognitive decline, and an enhanced effect of the strongest genetic risk factor for AD, the apolipoprotein E ε4 allele (*APOE*-ε4). Testosterone, largely regarded as a “male” sex hormone, has demonstrated protective effects against AD in rodent studies including both sexes. However, human research often only includes males, limiting our understanding of testosterone's effect on AD risk and cognitive function. In this study, we investigated whether testosterone levels in the blood relate to cognitive performance measuring overall (global) cognition, verbal memory (remembering word lists or stories), executive function (complex thinking/multitasking), processing speed (how quickly one completes thinking tasks), and language (naming objects) in both sexes. We also tested whether this relationship is influenced by the *APOE*-ε4 genetic risk factor. We found that in females carrying *APOE*-ε4, lower testosterone levels related to worse performance on global cognition, processing speed, and verbal memory tests; however, testosterone levels did not relate to cognitive performance on any test in males nor in females without the *APOE*-ε4 genetic risk factor. Our findings suggest that the lower testosterone levels may be a contributing factor to worse AD outcomes in women, particularly for those at higher genetic risk for AD. Our results also demonstrate the importance of including female participants and considering the *APOE*-ε4 genetic risk factor when studying testosterone and brain health.

**Supplementary Information:**

The online version contains supplementary material available at 10.1186/s13293-024-00620-4.

## Background

There are important sex differences in Alzheimer’s Disease (AD), the predominant form of dementia. Compared to men, women have a higher prevalence of AD diagnoses [1], show greater pathological burden in the early disease stages [2–6], and a different cognitive trajectory as the disease progresses [7, 8]. Women also demonstrate a more adverse effect of the apolipoprotein E ε4 allele (*APOE*-ε4), the most dominant genetic AD risk factor, on AD risk [9–11] and other AD-related outcomes [12, 13].

The mechanisms underlying these sex differences in AD are unknown, reflecting a knowledge gap that impedes optimizing treatments. However, sex differences often suggest a role of sex hormones. While many studies have examined how endogenous and exogenous levels of estrogen relate to brain health outcomes in women [14, 15], testosterone has been historically regarded as a male hormone and is often overlooked in women’s brain health research. Although circulating levels of testosterone in women are on average one tenth of that in men [16], women demonstrate a range of peripheral testosterone levels, and testosterone levels in the brain are similar between the two sexes [17, 18]. Furthermore, endogenous testosterone levels are close to, or even higher than, circulating estrogen levels in postmenopausal women [19]. Preclinical studies have demonstrated testosterone’s neuroprotective effects [20, 21], such as supplemental testosterone increasing spine synapses in the rat hippocampus [22], promoting neuronal plasticity via stimulating cellular differentiation [23], and reducing the reactivity of astrocytes from brain insults [24].

Low testosterone has been extensively studied as a risk factor for AD and cognitive deficits in older men [25–27]. Many studies have linked lower testosterone levels to higher AD risk [28, 29] and poorer cognitive function [30–32] in healthy aging men; however, results have not always been consistent [32–34]. Furthermore, the relationship of testosterone to the AD-specific pathologies of amyloid-beta and phospohorylated tau (p-Tau) has been studied in cellular and animal models. Testosterone treatment of cultured rat neurons reduced amyloid-beta precursor protein and soluble amyloid-beta secretion over time [35] and protected hippocampal neurons from amyloid-beta induced cell death [36]. In male rats with gonadectomies, testosterone treatment reversed amyloid-beta accumulation in the brain [37]. In regards to p-Tau, studies including male and female gonadectomized rats demonstrated that the removal of endogenous hormones causes tau hyperphosphorylation, but found a reversal of this effect following testosterone treatment [38, 39].

Both animal and human studies demonstrate that the effects of testosterone on the brain may be moderated by *APOE* genotype, likely because of a shared role of *APOE* and testosterone in the lipoprotein pathway and in inflammatory mechanisms [40], which in turn modulate androgen receptor sensitivity [41]. Pfankuch et al. [42] found that castration impaired novel location recognition in male *APOE*-ε4 mice, but not *APOE*-ε3 or *APOE* knockout mice. Raber et al. [43] found that blockade of androgen receptors in male mice led to memory deficits in *APOE*-ε4 mice only. Notably, the same study found that in female mice, androgen treatment improved cognitive outcomes in *APOE*-ε4 mice, but not in *APOE*-ε3 or *APOE* knockout mice. Therefore, animal models have highlighted the importance of examining the *APOE*-ε4 by testosterone interaction in both sexes. Human studies have also demonstrated an *APOE*-ε4 and testosterone interaction; however, the direction of this interaction in has been less consistent and women are typically not included in these studies. Specifically, some studies demonstrate that certain associations are stronger in *APOE*-ε4 male carriers, such as lower testosterone relating to smaller hippocampi [44] and poorer episodic memory [45], whereas another study reported that lower testosterone relates to higher likelihood of AD diagnosis in older male *APOE*-ε4 non-carriers only [46].

We previously analyzed participants of the Alzheimer’s Disease Neuroimaging Initiative (ADNI) and found that low plasma levels of total and free testosterone related to higher cerebrospinal fluid (CSF) p-Tau levels. When stratifying by sex assigned at birth, we found significantly higher levels of CSF p-Tau in *APOE*-ε4 carrier females versus males; however, this difference was eliminated after adjusting for testosterone levels [47]. These findings suggest that the lower testosterone levels in females compared to males may contribute to higher p-Tau biomarker levels and highlight the importance of including women in studies of testosterone. In the present study, we extended these findings and examine the interaction between testosterone and *APOE*-ε4 on clinical diagnostic group (cognitively normal (CN), mild cognitive impairment (MCI), and AD dementia (AD)) and global and domain-specific cognitive function in females versus males (sex assigned at birth) across the AD continuum. Based on our previous findings [47], we hypothesized that lower testosterone levels would relate to a clinical diagnosis of cognitive impairment (MCI or AD versus CNs) and poorer cognitive function across clinical diagnostic groups in both females and males. We also hypothesized that *APOE*-ε4 genotype would show a moderating effect in females only, with female *APOE*-ε4 carriers showing stronger relationships between testosterone and cognitive outcomes than non-carriers.

## Methods

### Participants

Data was downloaded from ADNI, which is publicly accessible at http://adni.loni.usc.edu. ADNI is a longitudinal, multi-site, cohort study that aims to combine neuroimaging, neuropsychological, clinical, and biomarker assessments to measure prognosis and pathogenesis of AD. The enrollment, clinical characterization, and inclusion/exclusion criteria for ADNI have been previously described [48]. This study included baseline data from participants in ADNI who had cognitive test data, plasma-based free testosterone levels, as measured by the Rules Based Medicine (RBM) Human Discovery Map panel, and *APOE* genotype data. The sample included 561 older adults aged 55–90 (213 females, 348 males based on self-reported sex assigned at birth), where 290 (52%) were *APOE*-ε4 carriers. The sample included 155 (28%) cognitively normal, 294 (52%) early and late mild cognitive impairment, and 112 (20%) AD dementia individuals.

### Testosterone

Total testosterone levels (ng/mL) in plasma were measured on the Luminex xMAP platform by Biomarkers Consortium Plasma Proteomics Project Rules-Based Medicine multiplex (http://www.rulesbasedmedicine.com) as part of a panel of 190 analytes related to a diverse array of human disease. A Box-Cox transformation was applied to raw assay values to normalize the distribution. Since sex hormone binding globulin (SHBG) is a protein that binds testosterone, SHBG (nmol/L) values from the same panel were used to calculate free, or bioavailable testosterone level, using total testosterone/SHBG × 100. A Pearson’s correlation demonstrated that free and total testosterone were significantly correlated (r = 0.99, p < 0.001). Descriptions of assay and normalization methods for this panel, developed on the Luminex xMAP platform by Rules-Based Medicine (RBM), can be found in “Biomarkers Consortium Plasma Proteomics Data Primer 02Aug2013 Final.pdf”, available for download at http://adni.loni.usc.edu/data-samples/access-data/. In young healthy volunteers, the lowest detectable dose for total testosterone was 0.17 ng/mL, the lower assay limit was 0.029 ng/mL, and the plasma range was 0.11–6.5 ng/mL. In our sample, 26 female participants (< 5% of the total sample) had readings below the least detectable dose; these values were imputed for the normalized testosterone levels using a value of half of the lowest detectable dose (0.085 ng/mL).

### Cognitive measures

The cognitive outcomes included tests to assess global cognition (Mini Mental Status Exam [MMSE]), verbal memory (Logical Memory-Delayed Recall [LM-DR]), executive function (Trail Making Test, Part B [TMTB]), processing speed (Digit Symbol Substitution Test [DSST]) and language (Boston Naming Test [BNT]). Higher scores on the MMSE, LM-DR, DSST and BNT reflect better performance while lower scores on the TMTB (second to complete task) reflect better performance. Due to a skewed distribution, the LM-DR and TMTB scores were log-transformed.

### Clinical diagnosis

Diagnosis of CN versus MCI was based on the Jak/Bondi actuarial diagnostic methods. Application of the Jak/Bondi diagnosis to ADNI produced more discernible cognitive phenotypes, more stable diagnoses, stronger associations with AD biomarkers, and better predication of progression to dementia compared to conventional diagnostic criteria [49]. Jak/Bondi diagnostic methods include six neuropsychological tests representing three cognitive domains: Trail-Making Tests A and B (psychomotor speed and executive function domain), Category Fluency and Boston Naming Test (language domain) and the Auditory Verbal Learning Test (AVLT) Delayed Recall and Recognition tests (episodic memory domain). An impaired score was defined as more than one standard deviation below the age-, education-, and sex-corrected normative mean. MCI diagnosis required impairment on two tests within a cognitive domain or one impaired score in each of the three cognitive domains. Diagnostic criteria for AD was based on the conventional criteria used in ADNI including: Mini Mental State Examination (MMSE) score of 20–26, Clinical Dementia Rating score of 0.5–1, and a diagnosis of probable AD dementia based on the NINCDS/ADRDA criteria [50].

### Statistical analyses

Statistical analyses were run using free testosterone levels, as free testosterone is more biologically relevant; however, analyses were repeated with total testosterone levels to examine how results changed. Participant characteristics, testosterone levels, and cognitive outcomes were compared by sex and *APOE*-ε4 status using independent-samples t-tests for continuous variables and Pearson’s chi-squared tests for categorical variables. We conducted analysis of covariance tests (ANCOVAs) to test for an interaction between clinical diagnostic group (CN, MCI, AD) and *APOE*-ε4 allele status (carrier versus non-carrier) on the dependent variable, testosterone, in sex-stratified samples. For our analyses with cognitive outcomes, we then conducted a series of linear regression models in sex-stratified samples with the independent variables of testosterone, *APOE*-ε4 status, and their interaction, and the dependent variable of cognitive test score. For all statistical models, considered covariates included age, education, body mass index (BMI), and self-reported history of cardiovascular disease, such as hypertension or high cholestserol. Any covariate that was not significant in the multivariable regression model at a *p* ≤ 0.10 threshold level was removed from the final model. Statistical analyses were performed in SPSS Version 28 and R Version 2023.06.1 + 524.

## Results

### Sample characteristics

See Table 1 for sample characteristics and variables of interest by sex and *APOE*-ε4 carrier status. There was no significant difference in the percentage of *APOE*-ε4 carriers between sex (*p* = 0.92). On average, the female participants were significantly younger (*p* = 0.03) and less educated than males (*p* < 0.001). Additionally, females had a lower prevalence of cardiovascular disease (*p* = 0.06) and significantly lower BMI (*p* < 0.001). As expected, total and free testosterone levels were significantly lower in females (*p*s < 0.001), but there were no significant sex differences in cognitive test performance except for in the language domain, with males scoring significantly higher on the BNT (*p* < 0.001).

**Table 1:** Sample characteristics by sex and *APOE*-ε4 carrier status

	Female	Male	p-value (es)^a^	Female (n = 213)			Male (n = 348)		
				*APOE*-ε4+ (n = 109)	*APOE*-ε4− (n = 104)	p-value, (es)^a^	*APOE*-ε4+ (n = 181)	*APOE*-ε4− (n = 167)	p-value, (es)^a^
Age, mean (SD)	74.0 (7.6)	75.4 (7.2)	**p = 0.03** (0.19)	72.4 (7.0)	75.6 (7.8)	**p = 0.002 **(0.43)	74.9 (6.7)	75.9 (7.7)	p = 0.18
Years of education, Mean (SD)	14.9 (2.9)	15.9 (3.1)	**p < 0.001 **(0.31)	14.5 (2.9)	15.4 (2.8)	**p = 0.02 **(0.14)	15.9 (3.1)	15.8 (3.2)	p = 0.72
*APOE*-ε4 carriers, n (%)	109 (51%)	181 (52%)	p = 0.92	–	–	–	–	–	–
White, n (%)	202 (95%)	328 (94%)	p = 0.25	101 (93%)	101 (97%)	p = 0.41	174 (96%)	154 (92%)	p = 0.29
Cognitive status, n (%)	–	–	p = 0.45	–	–	**p < 0.001**	–	–	**p < 0.001**
Cognitively normal	56 (26%)	99 (28%)	–	9 (8%)	47 (45%)	–	36 (20%)	63 (38%)	–
MCI	110 (52%)	184 (53%)	–	69 (63%)	41 (40%)	–	100 (55%)	84 (50%)	–
AD dementia	47 (22%)	65 (19%)	–	31 (29%)	16 (15%)	–	45 (25%)	20 (12%)	–
BMI^b^, mean (SD)	25.3 (4.2)	26.6 (3.7)	**p < 0.001 **(0.33)	25.0 (4.0)	25.6 (4.4)	p = 0.32	26.3 (3.6)	26.9 (3.84)	p = 0.14
Self-reported history of cardiovascular events, n (%)	146 (69%)	265 (76%)	p = 0.06	75 (69%)	71 (68%)	p = 0.99	138 (76%)	127 (76%)	p = 1.00
Raw plasma total testosterone values (ng/mL), mean (SD)^c^	0.70 (0.43)	2.99 (1.94)	**p < 0.001 **(1.45)	0.68 (0.42)	0.71 (0.45)	p = 0.64	3.24 (2.59)	2.76 (0.99)	**p = 0.03 **(0.25)
Normalized plasma total testosterone level (ng/mL)^d^, mean (SD)	− 0.35 (0.39)	0.43 (0.2)	**p < 0.001 **(2.66)	− 0.32 (0.35)	− 0.38 (0.43)	p = 0.23	0.41 (0.19)	0.45 (0.22)	**p = 0.03 **(0.23)
Normalized plasma free testosterone level (ng/mL)^e^, mean (SD)	− 19.1 (21.5)	25.2 (12.5)	**p < 0.001 **(2.69)	− 20.7 (23.3)	− 17.5 (19.6)	p = 0.29	23.6 (11.8)	27.0 (13.0)	**p = 0.01 **(0.28)
Global cognition (MMSE), Mean (SD)	26.4 (2.4)	26.6 (2.3)	p = 0.36	25.8 (2.3)	27.1 (2.4)	**p < 0.001** (0.55)	26.3 (2.4)	27.0 (2.2)	**p = 0.003** (0.32)
Verbal memory (log-transformed LM-DR)^f^, mean (SD)	0.58 (0.37)	0.62 (0.3)	p = 0.25	0.4 (0.3)	0.7 (0.4)	**p < 0.001** (0.80)	0.5 (0.3)	0.7(0.3)	**p < 0.001** (0.51)
Executive function (log-transformed TMTB)^f^, mean (SD)	2.1 (0.2)	2.1 (0.2)	p = 0.59	2.1(0.2)	2.0 (0.2)	**p = 0.03 **(0.31)	2.1(0.2)	2.1 (0.2)	p = 0.06
Processing speed (DSST), mean (SD)	36.7 (13.3)	35.5 (11.5)	p = 0.28	35.6 (13.0)	37.8 (13.5)	p = 0.21	34.5 (11.1)	36.5 (11.9)	p = 0.10
Langauge (BNT), Mean (SD)	24.4 (5.0)	25.8 (4.2)	**p < 0.001** (0.31)	24.3 (4.5)	24.5 (5.5)	p = 0.73	25.4 (4.1)	26.2 (4.3)	p = 0.08

Bold font text indicates a statistically significant group difference

*APOE-ε4* apolipoprotein E ɛ4 allele (+ = carrier, − = non-carrier), *MCI* mild cognitive impairment, *AD* Alzheimer’s disease, *MMSE* Mini-Mental State Examination, *LM-DR* Logical Memory-Delayed Recall, *TMTB* Trail Making Test Part B, *DSST* Digit Symbol Substitution Test, *BNT* Boston Naming Test

^a^es = effect size; effect sizes are provided for significant differences; Absolute value of Cohen’s *d* was calculated for mean differences (0.2 = small, 0.5 = medium, 0.8 = large), and a phi coefficient is provided for differences in proportions (0.1 = small, 0.3 = medium, 0.5 = large)

^b^Height and weight were measured at baseline and body mass index (BMI) was calculated by dividing weight (kg) by height (m^2^)

^c^26 female participants had testosterone levels below the lowest detectable dose; these values were imputed for the normalized testosterone levels using a value of half of the lowest detectable dose

^d^Raw total testosterone assay values were normalized using Box-Cox transformation

^e^Free testosterone levels were calculated using normalized total testosterone/SHBG × 100

^f^Verbal Memory and Executive Function scores were log transformed

Across both sexes, *APOE*-ε4 carriers had a significantly higher prevalence of MCI and AD dementia diagnoses and lower mean scores on the MMSE and LM-DR than non-carriers (*p*s < 0.005). Female *APOE*-ε4 carriers were significantly younger (*p* = 0.002), less educated (*p* = 0.02) and had a lower mean score on the TMTB (*p* = 0.03) than female non-carriers. Male *APOE*-ε4 carriers had significantly lower plasma total testosterone (*p* = 0.03) and free testosterone (*p* = 0.01) compared to male non-carriers.

### Clinical diagnosis

**Fig. 1:**
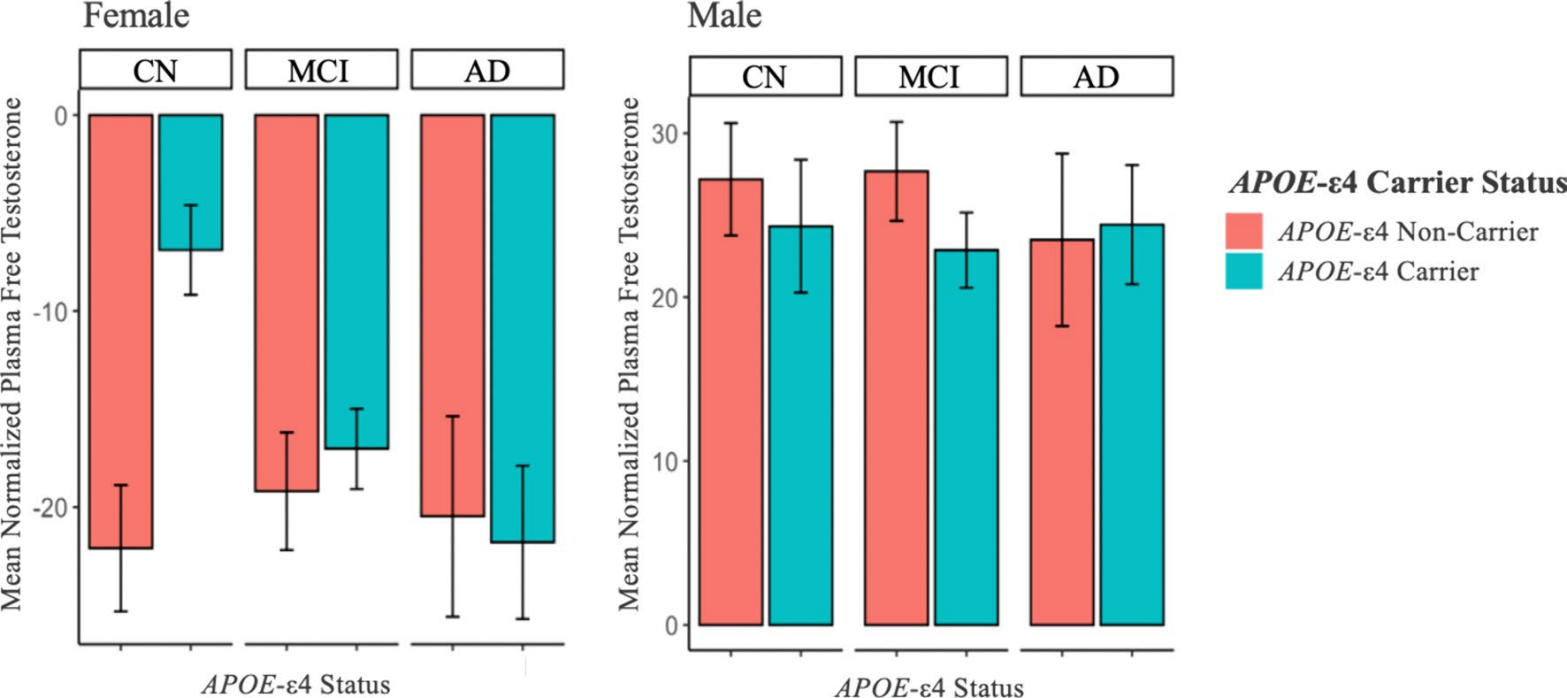
Mean free testosterone levels by clinical diagnostic group and *APOE*-ε4 status in both sexes. Using analysis of covariance (ANCOVA), we found no significant interactive effects of free testosterone by *APOE*-ε4, nor main effects of testosterone, in either sex, on clinical diagnostic group. Relevant covariates were age, education, BMI, and self-reported history of cardiovascular events. *CN* cognitively normal, *MCI* mild cognitive impairment, *AD* Alzheimer’s Dementia. Error bars represent standard error

The relationship of the clinical diagnostic group, and its interaction with *APOE*-ε4, to free testosterone levels in each sex was assessed. Among females, the main effects of clinical diagnostic group (F(2,209) = 0.46, *p* = 0.63), *APOE*-ε4 (F(1,209) = 1.21, *p* = 0.27), or their interaction (F(2,207) = 1.44, *p* = 0.24) on free testosterone levels were not significant. However, visual inspection of the data showed that, among female *APOE*-ε4 carriers only, free testosterone levels decreased as clinical diagnosis severity increased (Fig. 1). Female *APOE*-ε4 carriers showed higher free testosterone levels than female *APOE*-ε4 non-carriers in the CN and MCI groups, with this difference being significant and of moderate effect size within the CN group (t(13.4) = − 2.38*, p* = 0.03; Cohen’s d = − 0.80).

In males, the clinical diagnostic group by *APOE*-ε4 interaction was not significant (F(2,341) = 0.86, *p* = 0.43). After removing the interaction from the model, there was no main effect of clinical diagnostic group (F(2,343) = 0.26, *p* = 0.77), but, as expected based on sample descriptives, there was a main effect of *APOE*-ε4 (F(1,343) = 6.63, *p* = 0.01) on testosterone levels in males such that male *APOE*-ε4 carriers had significantly lower free testosterone levels than non-carriers, in contrast to the female CN group. Results did not substantively change when examining total testosterone levels.

### Global cognition

**Fig. 2:**
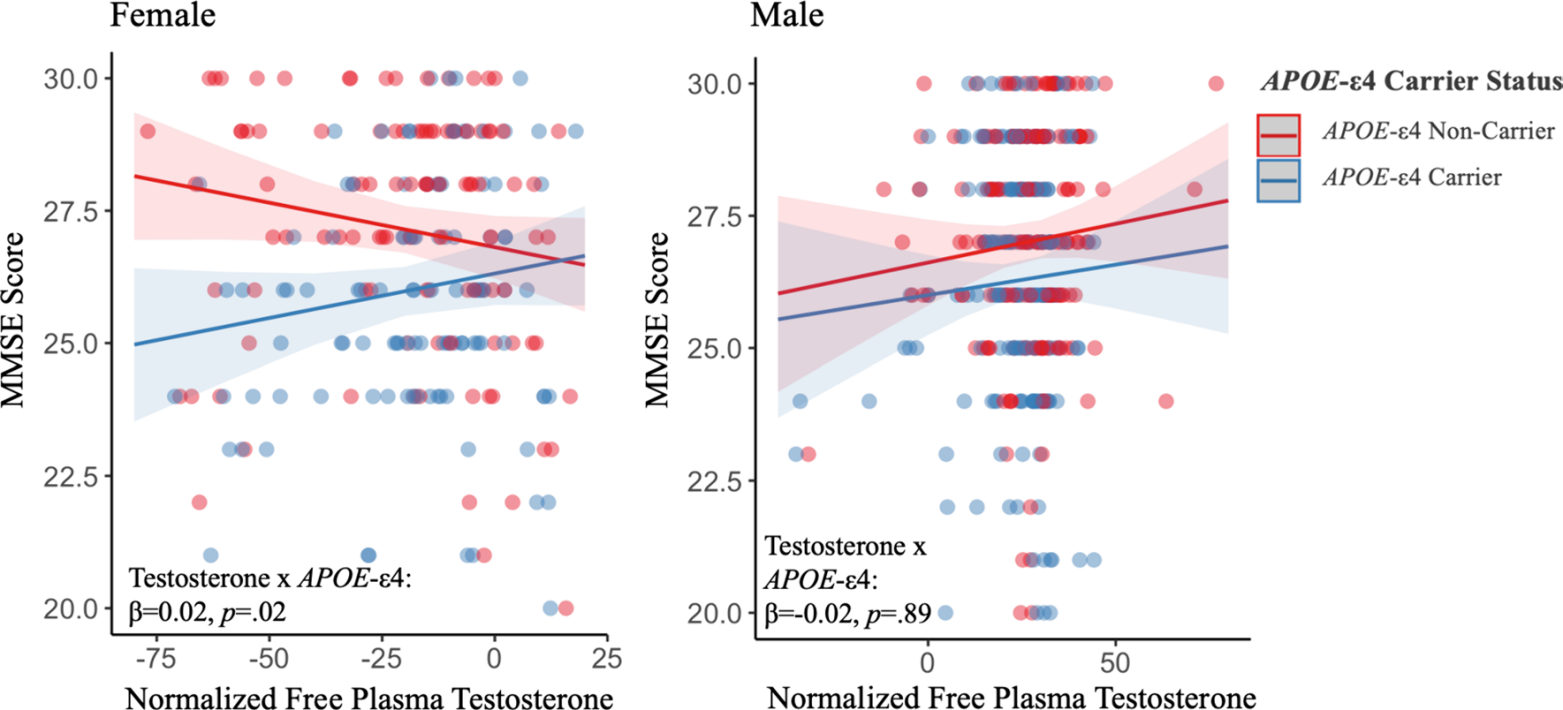
The relationship between freetestosterone levels and global cognition by *APOE*-ε4 carrier status in both sexes. Using multivariable linear regression, we revealed a significant freetestosterone by *APOE*-ε4 interaction and global cognition in female *APOE*-ε4 carriers only. In males, there were no significant effects of free testosterone levels nor its interaction with *APOE*-ε4 on global cognition. Relevant covariates were age, education, BMI, and self-reported history of cardiovascular events. The p-value and beta coefficient for each test are included

The relationship between free testosterone and global cognition (MMSE) in each sex was assessed (Fig. 2). In females, the linear model revealed a significant testosterone by *APOE*-ε4 interaction (β = 0.23, SE = 0.001, *p* = 0.02). In female *APOE*-ε4 carriers, there was a trending relationship between higher testosterone levels and better MMSE performance (β = 0.14, SE = 0.001, *p* = 0.10), whereas this trend was reversed in female *APOE*-ε4 non-carriers, (β = − 0.17, SE = 0.001, *p* = 0.09). In males, there was no significant or trending testosterone by *APOE*-ε4 interaction (β = − 0.02, SE = 0.002, *p* = 0.89) or main effect of testosterone (β = 0.08, SE = 0.001, *p* = 0.29) on MMSE performance. Results did not substantively change when examining total testosterone levels.

Due to the unexpected lack of a relationship between testosterone levels and global cognition in men, we conducted a sensitivity analysis to examine whether this relationship would manifest among a subset of men in the lowest tertile of normalized free testosterone levels (≤ 20.18 ng/mL). Eighty-seven (25%) of men were in this low testosterone group (see Supplemental Table 1 for sample characteristics by *APOE*-ε4 status in this male subset). In males with low testosterone levels, there was no significant or trending testosterone by *APOE*-ε4 interaction (β = 0.17, SE = 0.04, *p* = 0.38), nor main effect of testosterone (β = 0.20, SE = 0.03, *p* = 0.25). Results did not substantively change when examining total testosterone levels.

### Verbal memory

**Fig. 3:**
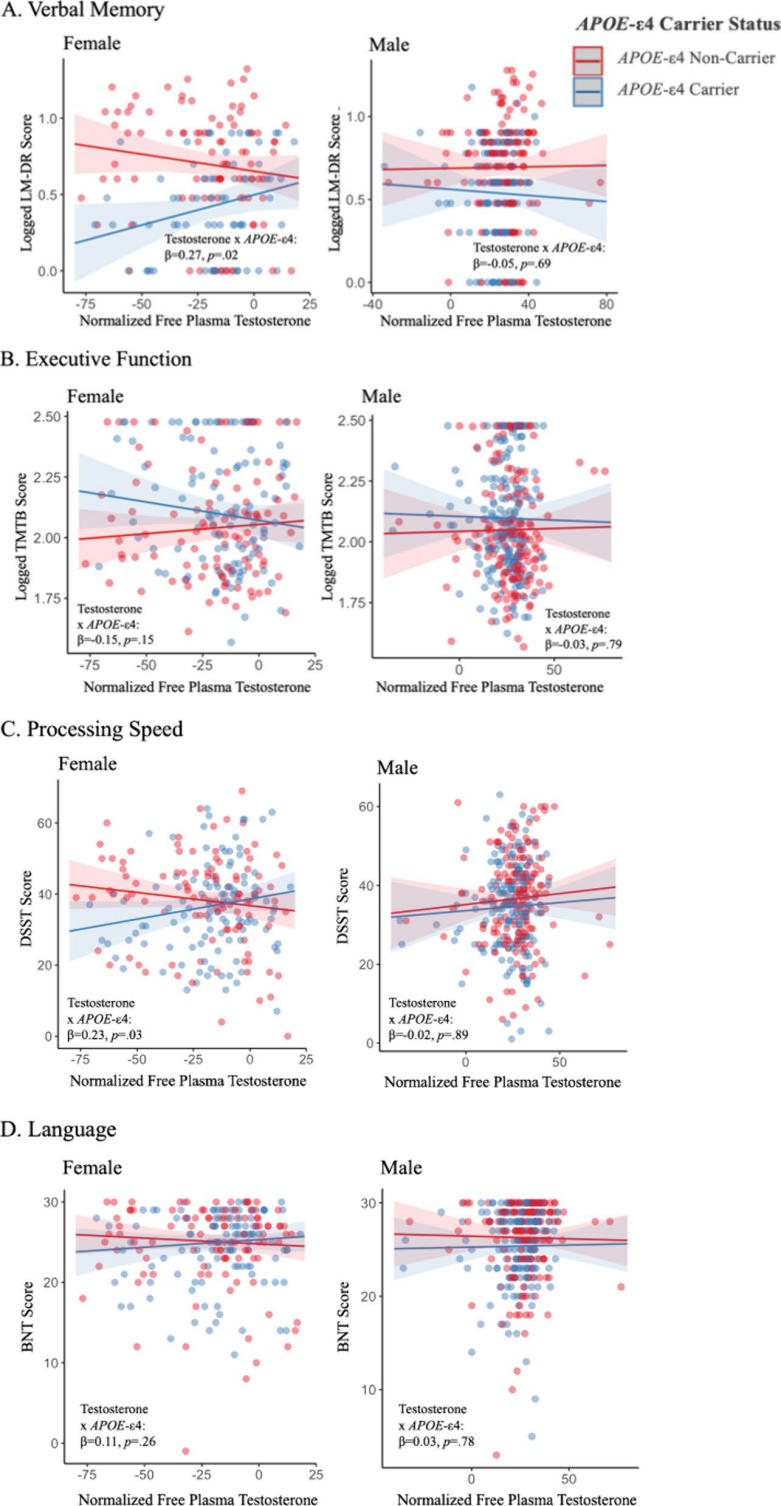
The relationship between freetestosterone levels and domain-specific cognitive function by *APOE*-ε4 carrier status in both sexes. Multivariable linear regressions were performed to test for a free testosterone by *APOE*-ε4 interaction on domain-specific cognitive outcome in each sex. **A**, **C** Show a significant testosterone by *APOE*-ε4 interaction on verbal memory (**A**) and processing speed (**C**) in female *APOE*-ε4 carriers only. In males, there were no significant effects of free testosterone, nor its interaction with *APOE*-ε4 on the domain-specific cognitive outcomes in **A** or **C**. **B**, **D** Show no significant testosterone by *APOE*-ε4 interactions on executive function (**B**) and language (**D**) in either sex. Relevant covariates were age, education, BMI, and self-reported history of cardiovascular events. The p-value and beta coefficient for each test are included

The relationship between free testosterone and verbal memory (LM-DR) in each sex was assessed (Fig. 3A). The linear model revealed a significant testosterone by *APOE*-ε4 interaction in females (β = 0.27, SE = 0.003, *p* = 0.02). In female *APOE*-ε4 carriers, higher testosterone levels significantly related to better LM-DR performance (β = 0.26, SE = 0.002, *p* = 0.03). This relationship was reversed in female *APOE*-ε4 non-carriers, although it was not significant nor trending (β = − 0.14, SE = 0.002, *p* = 0.19). In males, there was no significant or trending testosterone by *APOE*-ε4 interaction (all: β = − 0.05, SE = 0.003, *p* = 0.69) nor main effect of testosterone (β = 0.00929, SE = 0.002, *p* = 0.90) on LM-DR performance. Results did not substantively change when examining total testosterone levels.

In a sensitivity analysis limited to men in the lowest tertile of free testsosterone values, there was no significant or trending testosterone by *APOE*-ε4 interaction (β = − 0.08, SE = 0.006, *p* = 0.71), nor main effect of testosterone (β = 0.02, SE = 0.004, *p* = 0.92) on LM-DR performance. Results did not substantively change when examining total testosterone levels.

### Executive function

The relationship between free testosterone and executive function (TMTB) in each sex was assessed (Fig. 3B). The linear model revealed no significant or trending testosterone by *APOE*-ε4 interaction in females (β = − 0.15, SE = 0.002, *p* = 0.15) or males (β = − 0.03, SE = 0.002, *p* = 0.79), nor any significant or trending main effect of testosterone in females (β = 0.07, SE = 0.001, *p* = 0.45) or males (β = 0.01, SE = 0.001, *p* = 0.86). Results did not substantively change when examining total testosterone levels.

In a sensitivity analysis limited to men in the lowest tertile of free testsosterone values, there was no significant or trending testosterone by *APOE*-ε4 interaction (β = − 0.22, SE = 0.004, *p* = 0.29), nor main effect of testosterone (β = 0.11, SE = 0.003, *p* = 0.54) on TMTB performance. Results did not substantively change when examining total testosterone levels.

### Processing speed

The relationship between free testosterone and processing speed (DSST) in each sex was assessed (Fig. 3C). The linear model revealed a significant testosterone by *APOE*-ε4 interaction in females (β = 0.23, SE = 0.09, *p* = 0.03). In female *APOE*-ε4 carriers, higher free testosterone levels was trending in relation to better DSST performance (β = 0.17, SE = 0.06, *p* = 0.08), whereas this relationship reached significance when examining total testosterone (β = 0.19, SE = 0.42, *p* = 0.05). This association was reversed in female ε4 non-carriers, although it was not significant nor trending (β = − 0.13, SE = 0.06,* p* = 0.20). In males, there were no significant or trending testosterone by *APOE*-ε4 interaction (β = − 0.02, SE = 0.10, *p* = 0.89) or main effect of testosterone (β = 0.06, SE = 0.07, *p* = 0.42) on DSST performance. Aside from the result in female *APOE*-ε4 carriers, results did not substantively change when examining total testosterone levels.

In a sensitivity analysis limited to men in the lowest tertile of free testsosterone values, there was no significant or trending testosterone by *APOE*-ε4 interaction (β = 0.16, SE = 0.20, *p* = 0.42), nor main effect of testosterone (β = 0.07, SE = 0.16, *p* = 0.68) on DSST performance. Results did not substantively change when examining total testosterone levels.

### Language

The relationship between free testosterone and language (BNT) in each sex was assessed (Fig. 3D). The linear model revealed no significant or trending testosterone by *APOE*-ε4 interaction in females (β = 0.11, SE = 0.03, *p* = 0.26) or men (β = 0.03, SE = 0.04, *p* = 0.78), nor any significant or trending main effect of testosterone in females (β = − 0.06, SE = 0.02, *p* = 0.46) or in males (β = − 0.02, SE = 0.03, *p* = 0.83). Results did not substantively change when examining total testosterone levels.

In a sensitivity analysis limited to men in the lowest tertile of free testsosterone values, there was no significant or trending testosterone by *APOE*-ε4 interaction (β = 0.16, SE = 0.09, *p* = 0.44), nor main effect of testosterone (β = − 0.17, SE = 0.07, *p* = 0.35) on BNT performance. Results did not substantively change when examining total testosterone levels.

## Discussion

Our findings demonstrate that, among older female *APOE*-ε4 carriers, higher circulating free testosterone levels related to better global cognition, verbal memory, and processing speed scores. In contrast, these relationships were not observed among female non-carriers, nor in males, even when limiting to a low testosterone male sample. These relationships in females were domain-specific, as they were not observed with executive function or language performance. The specificity of the testosterone and cognition relationship to female *APOE*-ε4 carriers is consistent with our hypotheses and with our prior findings of lower testosterone levels relating to higher CSF p-Tau levels, particularly among female *APOE*-ε4 carriers [47]. However, inconsistent with our hypotheses, testosterone did not relate to cognitive outcomes in males, regardless of *APOE*-ε4 genotype.

Also contrary to hypotheses, testosterone levels did not differ by clinical diagnostic group, regardless of sex or *APOE*-ε4 genotype. These inconsistencies are likely due to the domain-specificity of the testosterone and cognition relationships in females, as multiple domains factored into the clinical diagnosis, and may be a consequence of the lower statistical power when analyzing relationships with categorical variables, such as diagnostic group, versus continuous data, such as testosterone levels. Studies with larger samples across the healthy aging, MCI, and AD trajectory are needed to more definitively test testosterone by *APOE*-ε4 interactions on clinical diagnostic group. Overall, these results add to our prior findings in support of a role of testosterone in postmenopausal women’s brain health and further underscore the importance of examining testosterone in women as opposed to limiting testosterone studies to men.

As mentioned in the introduction, testosterone has neuroprotective effects [20, 22–24] including those specifically against AD pathology [35–39], lending biological plausibility to our findings of higher testosterone relating to better cognition across the AD continuum. Another possibility is that the observed testosterone and cognition links in females may be driven by estrogen related mechanisms. Whereas ovarian production of estradiol decreases substantially during menopause, testosterone levels decline gradually throughout adulthood in females, similar to males [17, 51]. Circulating estrogens are also synthesized by the aromatization of testosterone via the enzyme aromatase [52, 53]. Previous studies in postmenopausal women have demonstrated that aromatase mRNA levels in adipose tissue increase as a function of age [54], reflecting an increase in the conversion of testosterone to estrogen. In fact, after the menopause transition, the aromatization of androgens in peripheral tissues becomes the primary source of estrogen production in postmenopausal females [55–57]. Like testosterone, estrogen has neuroprotective effects [58], including stimulating neurogenesis and synaptogenesis [59–61] and protecting against amyloid-beta plaque deposition [62]. Another similarity to testosterone is evidence of an estrogen and *APOE*-ε4 interaction, where the deleterious effects of postmenopausal estrogen deficiency on the brain appear more robust in *APOE*-ε4 carriers compared to non-carriers [63–65]. Levels of circulating estradiol were not available ADNI, which precluded our ability to consider the role of estrogen in the present study, but examining both sex hormones and their relationship with *APOE*-ε4 may provide a clearer picture for our sex-specific results.

The relationships between testosterone and cognition in females were limited to the cognitive outcomes of global cognition, verbal memory and processing speed. On average, women tend to show better performance on these cognitive domains over men [66–69]. The female advantage in verbal memory has been studied in the context of the AD continuum and is found to persist in the early AD stages [70–72]. Cognitive sex biases suggest that sex hormones may play a role in brain development and functioning related to these cognitive domains. Accordantly, studies have shown that performance on verbal memory and processing speed tests worsen during the menopausal transition [73, 74], depend on estrogen receptor genotype [75], and decline with increasing years since menopause [76]. In conjuction with these past findings, the specificity of our findings to cognitive domains with a female bias may further support a mechanistic role of the aromatization of androgens to estrogen in explaining our results. Although few studies have examined testosterone and cognitive links in women, our findings differ from some previous results. Hogervorst et al. [46] found a negative relationship between testosterone levels and processing speed performance and Yaffe et al. [77] found that testosterone levels did not relate to MMSE performance in older women. Our findings suggest that these inconsistencies may be due to a lack of accounting for the moderating role of *APOE*-ε4 status in these prior studies.

The lack of a relationship between testosterone levels and cognition in males was inconsistent with our hypotheses, and we can only speculate as to why we obtained this null result. One possibility is that there was a greater variance of testosterone levels in females compared to males, with the standard deviation of free and total testosterone in females double the male standard deviations. The larger spectrum of female testosterone values allows more opportunity to observe how differing testosterone levels relate to cognition. Another possibility may relate to our cognitive outcomes, as previous work has demonstrated that testosterone selectively relates to visuospatial performance in older men [78–81], and our present analysis did not include a visuospatial task. In addition, other studies have found a null association between testosterone treatment and cognitive outcomes when studying men who are within the low-to-normal range of testosterone, rather than hypogonadal or severely androgen-deficient [82, 83]. Studies aiming to characterize hypogonadism in the aging male population have found that symptoms often do not map onto plasma testosterone levels, due to normal age-related testosterone decline [84, 85]. Thus, the field has moved towards diagnosing hypogonadism in older men only if clinical symptoms are present in conjunction with low testosterone values [86, 87]. Due to the lack of clinical symptom data available in ADNI, we elected to use a tertile split to conduct sensitivity analysis specifically among male participants in the lowest normalized free testosterone values. We continued to not detect significant associations between cognitive outcomes and testosterone levels, nor the testosterone by *APOE*-ε4 interaction, in this subset; however, it remains unknown whether results would vary if we were able to clinically characterize hypogonadal males.

Our results suggest a contributing role of low testosterone levels in cognitive decline in female *APOE*-ε4 carriers and, thus, stress the importance of considering *APOE*-ε4 status when examining the role of testosterone in brain health, particularly in women. Additionally, our results suggest a potential mechanism underlying the stronger effect of *APOE*-ε4 in females on AD risk and AD-related outcomes. Testosterone levels are commonly lower in females compared to males, and if the *APOE*-ε4 allele has a more adverse effect on cognition in the context of lower testosterone levels, then more females may be susceptible to this genetic effect. Another explanation could be that testosterone may have a threshold amount needed to support the brain’s cognitive functioning during aging, especially in those with *APOE*-ε4 alleles, and males may not fall below this threshold even as their testosterone levels decline with age. Even among men in the lowest quartile of free testosterone levels, the average testosterone level was still higher than in our female sample, suggesting that even the lower range of testosterone values in men may not be deficient enough to exacerbate the negative effects of *APOE*-ε4 on cognition. However, it is again unknown how results might differ among a clinically characterized hypogonadal male sample.

Multiple biological pathways have linked testosterone levels to *APOE* genotype. First, circulating testosterone is highly implicated in high density lipoprotein cholesterol metabolism [88, 89], and *APOE* is a major transporter of lipoproteins in the brain [90], suggesting interactions between testosterone and *APOE* genotypes in metabolic brain pathways. Secondly, *APOE*-ε4 and testosterone both have links to inflammation. In addition to neuroprotective effects, testosterone has been shown to have antinflammatory effects [91–93] in both sexes. Conversely, experimental animal models suggest that the *APOE*-ε4 genotype is associated with a proinflammatory response [94, 95]. Thus, *APOE*-ε4 carriers may benefit more strongly from testosterone’s anti-inflammatory effects or the combination of low testosterone levels and the *APOE*-ε4 genotype may increase neuroinflammation. Lastly, the *APOE*-ε4 allele is associated with reduced amounts of androgen receptors in the neocortex [43], suggesting that *APOE*-ε4 carriers may have amplified effects from low testosterone due to weaker androgen signaling.

There are several study limitations that warrant consideration. First, the largely white and well-educated sample available through the ADNI database is not reflective of the overall United States population. Studies are needed in more diverse, community-based cohorts to examine the generalizability of our results. Secondly, a lack of available plasma estradiol data in the ADNI database precluded us from examining whether these effects were estrogen-independent or mediated by the aromatization of testosterone to estrogen. Also, there were no available CSF measures of testosterone, which would have better measured brain levels of the sex hormone and likely been more relevant for examining the effect of testosterone on cognitive functioning. While we conducted a sensitivity analyses among men in the lowest tertile of testosterone levels, we were unable to test prior findings of a testosterone and cognition relationship among hypogonadal men, due to the unavailability of hypogonadal clinical symptom data in ADNI. Additionally, this study was cross-sectional so we were not able to determine the temporal relationships between changes in testosterone and changes in cognition. Although evidence from prior studies suggests that testosterone’s effects precede AD outcomes, there is potential for bidirectional relationships where AD pathology may negatively feedback testosterone levels by inhibiting steroid hormone production [96, 97]. Lastly, we are unable to account for all potential confounding variables, such as medications taken that may influence both sex hormone production and cognitive performance. Despite these limitations, our findings contribute to the understanding of sex differences in cognitive performance across the healthy aging and AD continuum and underscore potential for missed scientific discoveries when analyses involving testosterone are limited to men.

## Perspectives and significance

Our findings hold clinical significance in that low testosterone levels are potentially modifiable. Although numerous studies have investigated whether testosterone supplementation has an effect on cognition [98] and AD pathology [99] in men, the benefits of testosterone supplementation in relation to older women’s cognitive health remain largely uninvestigated, although testosterone therapy has recently been studied in women’s sexual health [100]. However, testosterone modulation may be achieved without supplementation, as testosterone is one of the primary molecules secreted during resistance exercise [101] in men and women [102, 103]. Our results may suggest that either pharmaceutical or lifestyle approaches to increasing low testosterone levels may be particularly beneficial for cognitive health in female *APOE*-ε4 carriers. Furthermore, our results may enlighten mechanisms underlying cognitive sex difference and the more adverse effect of the *APOE*-ε4 allele in female carriers and therefore emphasize the importance of including women and accounting for *APOE*-ε4 status when examining testosterone and brain health. While our findings require replication in more generalizable cohorts and with longitudinal data, our findings underscore the potential for missed scientific discoveries when analyses involving testosterone are limited to men.

## Conclusions

In conclusion, our results suggest that female *APOE*-ε4 carriers are most sensitive to testosterone’s effects on cognitive performance. We demonstrated that in female *APOE*-ε4 carriers, lower plasma testosterone was associated with worse global cognition, processing speed, and verbal memory, but found no associations between testosterone and cognition in female *APOE*-ε4 non-carriers, nor in men, regardless of *APOE*-ε4 status or whether testosterone levels were in the low range for males. These analyses underscore the importance of considering sex and *APOE*-ε4 status in testosterone and brain health studies, and posits potential for modifiable risk factors for cognitive decline, particularly among women at higher genetic risk for AD.

### Supplementary Information

Supplementary Material 1.

## Data Availability

The dataset supporting this article is available in the ADNI repository [adni.loni.usc.edu].
