# Supplementary material for: Low testosterone levels relate to poorer cognitive function in women in an APOE-ε4-dependant manner
Source: Biol Sex Differ. 2024 Jun 5;15:45. doi: 10.1186/s13293-024-00620-4 (PMC11151480; doi:10.1186/s13293-024-00620-4)
Supplement: Supplementary file 1 — Supplementary Material 1. [file 13293_2024_620_MOESM1_ESM.docx]

| Supplemental Table 1. Sample characteristics of hypogonadal male subset | | | | |
| --- | --- | --- | --- | --- |
|  | Hypogonadal Males (n=87) | | | |
|  | Overall | *APOE*- ε4 + (n=53) | *APOE*-ε4 - (n=34) | p-value, (es)^a^ |
| Age, Mean (SD) | 78.1 (6.6) | 76.6 (6.5) | 80.5 (6.2) | **p=.006**  (0.62) |
| Years of education, Mean (SD) | 16.0 (3.0) | 16.4 (2.7) | 15.3 (3.3) | p=.10 |
| White, n (%) | 83 (95%) | 51 (96%) | 32 (94%) | p=.51 |
| Cognitive status, n (%) | - | - | - | p=.08 |
| Cognitively normal | 27 (31%) | 12 (23%) | 15 (44%) | - |
| MCI | 43 (49%) | 28 (53%) | 15 (44%) | - |
| AD dementia | 17 (20%) | 13 (24%) | 4 (12%) | - |
| BMI^b^, Mean (SD) | 25.9 (3.9) | 25.9 (3.7) | 25.8 (4.2) | p=.95 |
| Self-reported history of cardiovascular events, n (%) | 70 (80%) | 46 (87%) | 24 (71%) | p=.11 |
| Raw plasma total testosterone values (ng/mL), Mean(SD) | 1.58 (0.52) | 1.61 (0.51) | 1.55 (0.53) | p=.60 |
| Normalized plasma total testosterone level (ng/mL)^c^, Mean (SD) | 0.17 (0.19) | 0.15 (0.20) | 0.18 (0.19) | p=0.62 |
| Normalized plasma free testosterone level (ng/mL)^e^, Mean (SD) | 9.60 (11.74) | 10.08 (12.15) | 8.84 (11.20) | p=0.63 |
| Global Cognition (MMSE), Mean (SD) | 26.7 (2.1) | 26.4 (2.4) | 27.2 (1.7) | p=0.09 |
| Verbal Memory (Log-transformed LM-DR)^e^, Mean (SD) | 0.62 (0.28) | 0.59 (0.28) | 0.67 (0.27) | p=0.24 |
| Executive Function (Log-transformed TMTB)^e^, Mean (SD) | 2.1 (0.2) | 2.07 (0.20) | 2.08 (0.23) | p=0.91 |
| Processing Speed (DSST), Mean (SD) | 35.2 (10.8) | 35.4 (9.9) | 34.8 (12.2) | p=0.82 |
| Langauge (BNT), Mean (SD) | 25.6 (4.7) | 25.4 (4.3) | 25.9 (5.4) | p=0.64 |

Note. Bold font text indicates a statistically significant group difference. ^a^es=effect size; effect sizes are provided for significant differences; Absolute value of Cohen’s *d* was calculated for mean differences (0.2 = small, 0.5 = medium, 0.8 = large), and a phi coefficient is provided for differences in proportions (0.1 = small, 0.3 = medium, 0.5 = large). ^b^Height and weight were measured at baseline and body mass index (BMI) was calculated by dividing weight (kg) by height (m^2^). ^c^26 female participants had testosterone levels below the lowest detectable dose; these values were imputed for the normalized testosterone levels using a value of half of the lowest detectable dose. ^d^Free testosterone levels were calculated using normalized total testosterone/SHBG × 100 ^e^Verbal Memory and Executive Function scores were log transformed; *APOE*-ε4 = apolipoprotein E ɛ4 allele ( + = carrier, - = non-carrier); MCI = mild cognitive impairment; AD = Alzheimer’s disease; MMSE = Mini-Mental State Examination; LM-DR = Logical Memory- Delayed Recall; TMTB= Trail Making Test Part B; DSST = Digit Symbol Substitution Test; BNT = Boston Naming Test.
